# Quantified temporal changes of heart rate variability when developing SIRS

**DOI:** 10.1186/cc11796

**Published:** 2012-11-14

**Authors:** N Matsumaru, K Shirai, Y Kawamura, Y Yokota, K Kumada, I Toyoda, S Ogura

**Affiliations:** 1 Advanced Critical Care Center, Gifu University Hospital, Gifu, Japan; 2 Faculty of Engineering, Gifu University, Gifu, Japan

## Background

Heart rate variability (HRV) has been reported to gradually decrease long before the onset of sepsis [1,2]. Since the development of systemic inflammatory response syndrome (SIRS) has been included in the definition of sepsis, we compared temporal changes of HRV depending on the SIRS state coming after.

## Methods

Vital data of patients admitted in ICU were downloaded from bedside monitoring systems. No specific registration criteria for disease were defined. Vital signs regarding SIRS evaluation - that is, hear rate, respiration rate, and core temperature - are recorded every 10 seconds, 0.1 Hz. By taking the median of 60 data, the SIRS state was determined for each hour, ignoring the count of white blood cells. The sampling rate of ECG data is 500 Hz, and the HRV for each minute (0.017 Hz) was calculated from that. Abnormal heartbeats and trends were eliminated using a stochastic model, and the power spectrum was derived with an autoregressive model [3]. We considered the integral value within 0.04 to 0.4 Hz as an estimate of HRV. Temporal linear trends of HRV were quantified as Spearman's correlation coefficient. Note that the target period for HRV trend evaluation is immediately before the SIRS evaluation period. The following software was used: IBM SPSS version 19, R version 2.15.0, and G*Power Version 3.1.4.

## Results

Forty-nine patients were registered, and 34 patients (69.4%) suffered from sepsis. The average age was 64.3 years, and the average ICU stay was 31.4 days, ranging from 3 to 113 days. The number of data where a SIRS state was identified was 21,919, and 56.5% (12,380) were evaluated as SIRS-positive. Immediately before SIRS-positive states, the mean value of the correlation coefficients was -0.00234 (SD 0.321). The mean value before SIRS-negative states was 0.0201 (SD 0.309). The mean difference was 0.0224 (*P *< 0.05), and the statistical power was 99.9%.

## Conclusion

Our analysis shows that the mean trend of the HRV value immediately before developing SIRS was negative, and the mean trend of HRV immediately before a non-SIRS state was positive. This result does support previous results. For sepsis monitoring, however, further investigation is mandatory. This result only suggests a stronger negative trend of HRV when developing SIRS for the next period, regardless of the current state.
